# Targeted Axillary Dissection in Node-Positive Breast Cancer: A Retrospective Study and Cost Analysis

**DOI:** 10.7759/cureus.14610

**Published:** 2021-04-21

**Authors:** Michèle Beniey, Kerianne Boulva, Samuel Rodriguez-Qizilbash, Ahmad Kaviani, Rami Younan, Erica Patocskai

**Affiliations:** 1 Department of General Surgery, Université de Montréal, Montreal, CAN; 2 Department of Surgical Oncology, Centre Hospitalier de l'Université de Montréal (CHUM), Montreal, CAN

**Keywords:** targeted axillary node dissection, breast cancer, breast cancer management, nodal metastases, neoadjuvant chemotherapy, radioactive seed, iodine seed, axillary lymph node dissection

## Abstract

Introduction

Targeted axillary dissection (TAD) is a novel technique in the field of surgical oncology. During TAD, patients with node-positive breast cancer who clinically responded to neoadjuvant chemotherapy undergo resection of a previously proven metastatic node together with sentinel lymph node dissection (SLND). We aimed to assess the success rates of seed insertion and seed retrieval in the Canadian setting, as well as hospital costs of the procedure.

Methods

Patients converted to clinically node-negative status post-neoadjuvant chemotherapy underwent TAD. Before surgery, an iodine-125 radioactive seed was inserted in the previously proven metastatic node. The seed node was resected together with an SLND. Axillary lymph node dissection (ALND) was performed in all patients with residual metastases.

Results

Radioactive seeds were successfully inserted in 34/35 patients. In 34 patients, the targeted node was successfully resected with the radioactive probe during TAD. In one patient, the seed was retrieved inferiorly in the axilla during surgery. There was no adverse event. In total, 50% (17/34) of patients had no residual metastases and were able to avoid ALND. Eight out of 17 patients who underwent ALND did not have any residual disease in their specimen. The mean cost of TAD was 25% superior to the mean cost of ALND (p = 0.02). However, the mean total cost of the hospital stay for TAD was 20% superior to the mean cost of ALND (p = 0.11). The mean cost of TAD was 4,322 Can$ (Canadian dollars), similar to the mean cost of both ALND and SLND performed during the same procedure (4,479 Can$).

Conclusions

TAD was successful in 97% of patients. Despite increased procedural costs, with a lesser impact on total hospital stay costs, TAD was beneficial in 50% of patients. These patients avoided the unnecessary morbidity associated with ALND.

## Introduction

Until recently, axillary lymph node dissection (ALND) was the standard technique used in lymph node-positive breast cancer. ALND improves locoregional control and provides key pathological information to guide post-operative treatment. Nevertheless, ALND is an invasive procedure associated with significant morbidity, and its prognostic role needs to be better defined.

Compared to sentinel lymph node dissection (SLND), ALND is associated with increased morbidity [1], higher rates of lymphedema, paresthesia, sensory loss in the arm, and impairment in shoulder function [2-5]. Patients undergoing SLND have fewer infections [4] and a better quality of life [2,3]. Over the past two decades, axillary dissection has been largely replaced by SLND in early-stage breast cancer. In patients with locally advanced breast cancer, neoadjuvant chemotherapy (NAC) followed by ALND is the standard management.

NAC results in pathologic complete response (pCR) in more than 40% of patients with axillary metastases [6,7]. Despite the improvement in pCR rates in breast cancer with the development of novel therapies, ALND is still considered a gold standard in most patients. Accurately identifying the subgroup of patients in whom ALND can be avoided remains a clinical challenge.

Previous studies have highlighted the limitations of SLND in accurately identifying patients with residual lymph node disease following chemotherapy [7-11]. In the ACOSOG Z1071 trial, the false-negative rate (FNR) of SLND post-chemotherapy was 13% in patients with stage T0-4, N1-2 M0 breast cancer [7]. High FNRs were also found in the SENTINA study, precluding the use of standard SLND in patients converted to clinically node-negative status (i.e. absence of palpable nodes or abnormal nodes on imaging) after the completion of chemotherapy [8]. These studies demonstrated that the use of a dual-tracer technique (i.e., both blue dye and a radioisotope) resulted in higher rates of sentinel lymph node identification [11]. Lower FNRs were achieved when at least two nodes were retrieved [8-10]. A decrease in the FNR was observed when a metallic marker was placed to retrieve the biopsy-proven positive node [7,10].

Targeted axillary dissection (TAD) is an innovative surgical procedure that emerged in an attempt to further decrease the FNR of SLND. In patients with axillary disease, a metallic marker is inserted in the suspicious node prior to neoadjuvant therapy. This allows for the targeted implantation of a radioactive iodine-125 seed after the completion of chemotherapy [12]. During TAD, the seed node is retrieved using a radioactive probe. The procedure is performed together with SLND using a dual-tracer technique [12,13]. An ALND is undertaken when one or more nodes are found metastatic on pathological analysis.

Our study has two aims. The first one is to evaluate the success rate and the procedural safety of seed insertion and seed node retrieval in the axilla in our Canadian setting. The second one is to assess the mean hospital cost of TAD and to compare it with costs of other common breast surgeries. Preliminary results of this article were presented at the 2017 Society of Surgical Oncology Annual Cancer Symposia and at the 39th Congress of the European Society of Surgical Oncology.

## Materials and methods

Study design and participants

**Figure 1:**
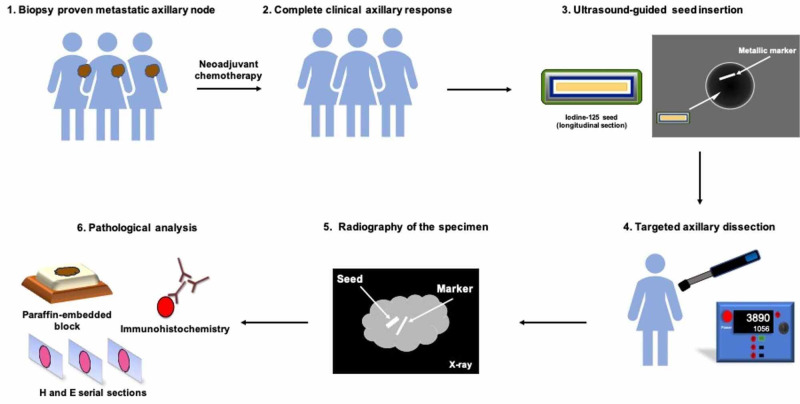
Main steps of the procedure.

We conducted a retrospective study at the University of Montreal Hospital Center. Data were collected from medical records in the hospital database between March 2016 and January 2019. Eligible patients were at least 18 years old and had proven invasive breast carcinoma and a biopsy-proven axillary metastasis with complete clinical axillary response to NAC (Figure 1). Complete clinical axillary response was determined during physical examination by a surgical oncologist and correlated with imaging for the entire cohort. All patients had a second examination by a surgical oncologist or a medical oncologist, as this is standard in our institution for patients under chemotherapy. We excluded patients who did not undergo TAD, had a radioactive seed placed in an axillary lymph node for another primary malignancy, did not receive NAC, had insufficient clinical and surgical data in their file, or underwent TAD with a single-tracer method. We used the STROBE (Strengthening the Reporting of Observational Studies in Epidemiology) Statement guidelines [14] for cohort studies to report this study. The study protocol was approved by the hospital’s institutional review board.

Iodine-125 seed insertion and surgical technique

All patients had a core needle biopsy or fine needle aspiration of abnormal nodes (i.e., increased diameter, cortical thickness, round shape, hilar compression, irregular margins) in radiology (Figure 1). An assessment was performed by a pathologist. A clip was inserted in the most prominent abnormal node, either during the diagnostic biopsy or after the node was proven metastatic. Patients received NAC and HER2 (human epidermal growth factor receptor 2) targeted therapy when HER2 was amplified in the breast biopsy specimen. For all patients, the iodine-125 seed was implanted under ultrasound guidance within five days of surgery. Radiography of the axilla was performed following seed insertion to ensure proper localization.

TAD, using the Neoprobe Gamma Detection System, was performed according to the previously published technique [15]. All surgeons used a dual-tracer technique, with preoperative retroareolar injection of both blue dye and technetium-99 [16]. All TAD procedures were performed together with breast surgery. Radiography of the axillary specimen was obtained in the operating room to confirm the presence of the metallic marker and radioactive seed prior to pathological evaluation (Figure 1).

Radioactive seed insertion was deemed successful when the iodine-125 seed was implanted in the targeted node and its position confirmed after the procedure. During TAD, seed node retrieval was deemed successful when the targeted node was removed with the seed using a radioactive probe. ALND was performed for proven lymph node metastases, including micrometastases and isolated tumor cells, and when TAD was unsuccessful.

Pathological analysis

In most cases (33/35), an intraoperative frozen section of all excised axillary lymph nodes was obtained. In all cases, a radioactive probe (Ludlum measurements Inc., Sweetwater, Texas, USA) was used to distinguish the iodine-125 seed from the metallic marker within the lymph node. All lymph nodes were paraffin-embedded. Serial 4-mm sections were obtained and stained with hematoxylin and eosin. Immunohistochemistry was performed in all cases. A breast pathologist evaluated the slides.

Cost analysis

Raw data were extracted from the hospital database by the financial resources’ directorate of our hospital center. In this analysis, we included hospital’s expenditures only. Among patients who underwent TAD, we obtained data for 33 out of 35 patients. No data were available for patients operated before April 2016. To identify the cost of TAD precisely, we excluded 13 patients who had TAD followed by ALND during the same procedure. In order to compare the cost of TAD with other common axillary surgeries, we extracted data from distinct patients who underwent ALND and SLND.

We compared costs among four different groups: 1) TAD (i.e., TAD alone), 2) ALND + SLND (i.e., SLND followed by ALND during the same procedure), 3) ALND (i.e., ALND alone), and 4) SLND (i.e., SLND alone). In our analysis, we included the cost of the procedure and the total cost of the hospital stay. The cost of the procedure includes costs of radiology and surgery departments on the day of surgery, as well as the cost of radioactive seed insertion. Costs of breast implants were excluded. The cost of the hospital stay includes the procedure, medication, laboratory analysis, multidisciplinary services (e.g., physiotherapy), administrative fees, hospital unit fees, or outpatient unit fees.

Statistical analysis

Using StataSE (StataCorp LP, College Station, TX, USA), we carried out descriptive statistics for patients’ baseline characteristics. For all hypothesis testing, the frequency distribution of each variable was analyzed first. To compare mean differences between the cost of TAD and the cost of ALND, we performed Mann-Whitney tests. For each analysis, confidence intervals were computed for a degree of 95% confidence. Statistical hypothesis testing was done with an alpha value of 0.05. Patients with missing information were excluded from the statistical analysis. We used the 8th edition of the AJCC (American Joint Committee on Cancer) manual for all stage classifications.

## Results

Characteristics of the cohort

**Figure 2:**
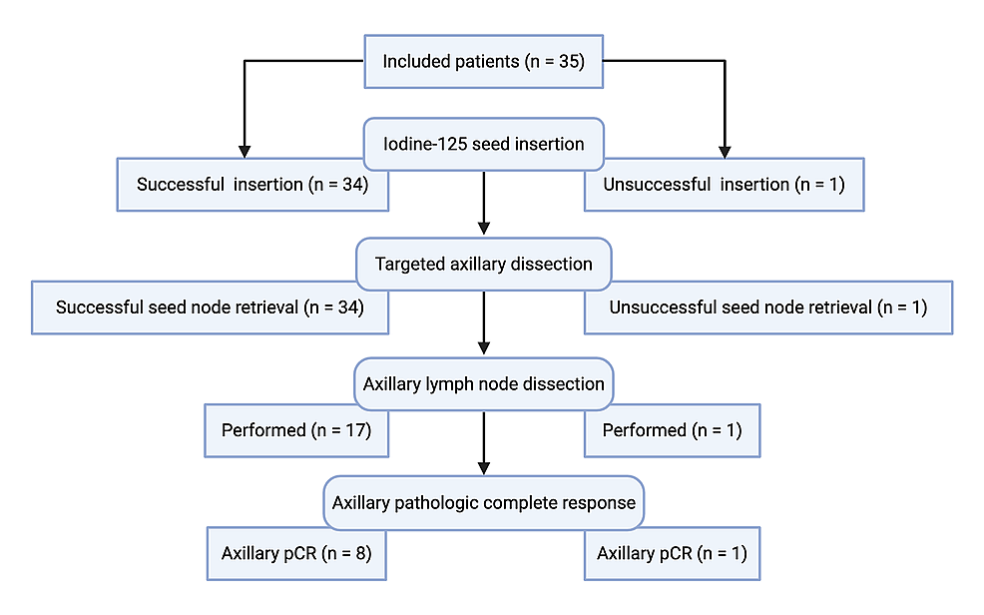
Flow diagram and procedural outcomes. pCR, pathologic complete response

A total of 35 women were included in the study (Figure 2). Participants’ clinicopathological characteristics are reported in Table 1. All patients received chemotherapy in the neoadjuvant setting. Out of 35 patients, 27 (77%) received an anthracycline-based regimen, two patients received carboplatin as well, two were part of an experimental research protocol, and four received other regimens. Four patients did not receive the entirety of their NAC regimen due to side effects and personal reasons. One out of four achieved pCR in the breast and two in the axilla. Eleven patients achieved pCR in both the breast and the axilla.

**Table 1:** Characteristics of the patients. *Invasive mixed carcinoma and invasive mammary carcinoma. **Tumor size on post-operative pathology report. AJCC, American Joint Committee on Cancer; HER2, human epidermal growth factor receptor 2; HR, hormone receptor; TAD, targeted axillary dissection

Characteristic	N	%
Age on day of TAD, years	Median: 49	Range: 29-76
Breast surgery		
Partial mastectomy	20	57.1
Skin-sparing mastectomy	11	31.4
Modified radical mastectomy	1	2.9
Total mastectomy	3	8.6
Histology pre-neoadjuvant chemotherapy		
Invasive ductal carcinoma	31	88.6
Invasive lobular carcinoma	1	2.9
Other*	3	8.6
Tumor size, cm**	Mean: 0.99	Range: 0-7
Clinical T stage (AJCC, 8th edition)		
T1c	6	17.1
T2	21	60.0
T3	7	20.0
T4d	1	2.9
Phenotype pre-neoadjuvant chemotherapy		
HR+/HER2-	21	60.0
HR+/HER2+	7	20.0
HR-/HER2+	3	8.6
Triple-negative	4	11.4
Clinical regional lymph nodes stage (AJCC, 8th edition)		
N1	32	91.4
N2	1	2.9
N3	2	5.7
Clinical prognostic stage (AJCC, 8th edition)		
IB	5	14.3
IIA	14	40.0
IIB	6	17.1
IIIA	4	11.4
IIIB	4	11.4
IIIC	2	5.7

Success rate and procedural safety

The implantation of the iodine-125 seed in the previously proven positive axillary node was successful in 34 out of 35 patients and no complications occurred (Figure 2). In two cases, no metallic marker was seen on ultrasound during seed insertion, and the radiologist localized the positive node using imaging characteristics (i.e., node size, localization, and morphology). On final pathology, one patient had complete response in both the breast and the axilla. The other patient underwent ALND due to residual disease in the seed node.

Overall, the radioactive iodine-125 seed was identified in all cases during surgery. The targeted node was resected uneventfully with the radioactive probe in 34/35 (97%) patients. In the remaining patient, the radioactive seed was retrieved inferiorly to the targeted node during surgery. In this patient, seed node retrieval was unsuccessful using the radioactive probe. However, the targeted node was identified as a sentinel node. An intraoperative radiography of the specimen revealed a metallic marker inside of the targeted node and confirmed its excision. This patient subsequently underwent ALND and adjuvant radiotherapy.

Of the 34 patients in whom TAD was completed, 17 (50%) had axillary pCR and did not require subsequent ALND (Figure 2). Interestingly, among the 17 patients in whom ALND was performed, no additional metastases were retrieved in eight patients. One adverse event was reported after TAD in a patient diagnosed with axillary wound cellulitis on post-operative day 9. The patient was readmitted and received a short course of intravenous antibiotics.

All patients had a complete clinical response on examination. On post-NAC imaging, in 21 patients (i.e., 21/34; 62% of the TAD cohort), the targeted node had a normal aspect or was decreased in size. Out of these 21 patients, 12 had residual metastases in their TAD specimen and underwent ALND. Twelve patients (i.e., 12/34; 35% of the TAD cohort) still had slightly abnormal radiographic features of the targeted node (i.e., thick cortex, irregular shape, hyperenhancement). Five of these 12 patients underwent ALND due to residual metastases.

Mean hospital costs

**Figure 3:**
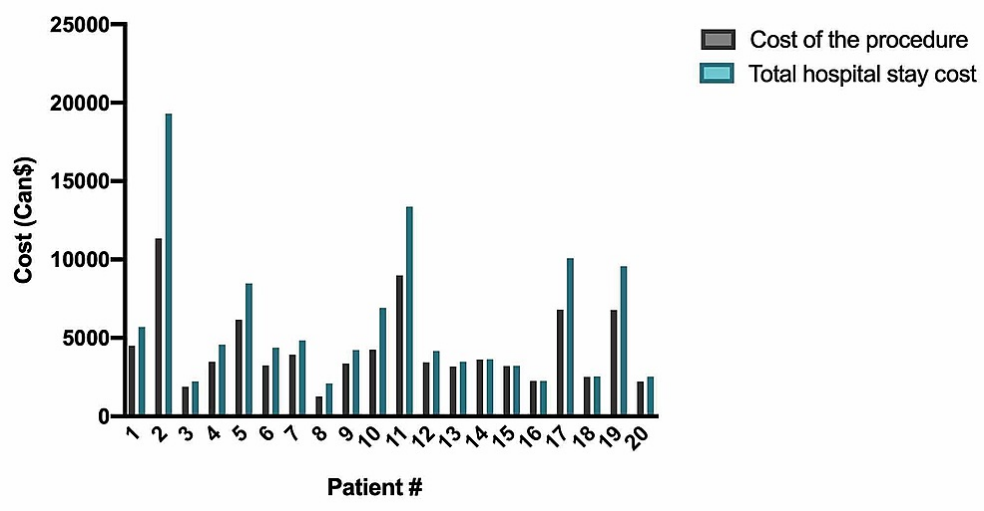
Costs distribution for targeted axillary dissection.

**Figure 4:**
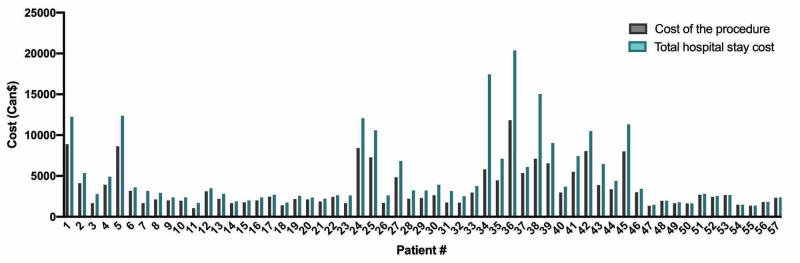
Cost distribution for axillary lymph node dissection.

**Figure 5:**
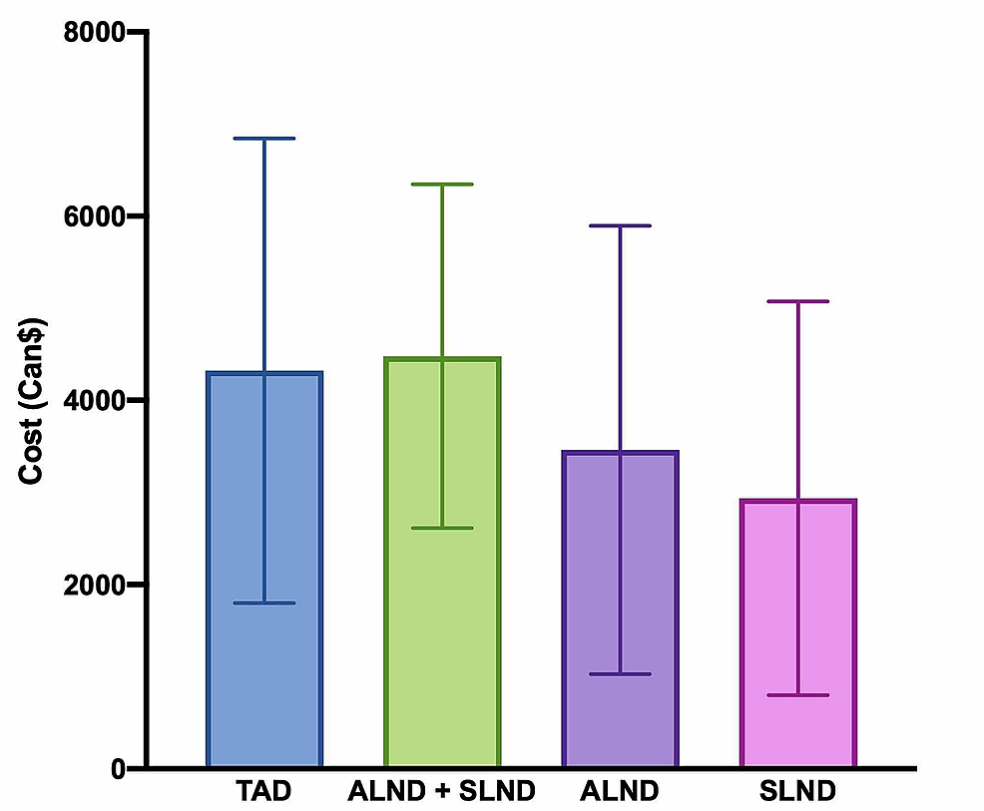
Mean procedural costs and standard deviations for targeted axillary dissection and other common axillary surgeries. ALND, axillary lymph node dissection; SLND, sentinel lymph node dissection; TAD, targeted axillary dissection

Our cost analysis includes 20 patients from our cohort who underwent TAD. In addition, we analyzed data of 57 patients who had ALND, 1,248 patients who had SLND, and 14 patients who had SLND followed by ALND during the same surgery. Figure 3 and Figure 4 depict the distribution of both procedural costs and total hospital stay costs for TAD and ALND, respectively. Despite differences in the number of patients in each group, we observed a similar distribution. We evaluated the cost of the procedure for TAD and several common axillary surgeries (Figure 5). We found that the mean cost of TAD in our cohort was 25% (860 Can$) superior to the mean cost of ALND (p = 0.02). Interestingly, the maximal cost for one patient was similar between the two groups: 11,349 Can$ for TAD and 11,829 Can$ for ALND. Furthermore, the mean total cost of the hospital stay for TAD was 20% (980 Can$) superior to the mean cost of ALND (p = 0.11).

## Discussion

Success rate and procedural safety

In this study, radioactive seeds were inserted and retrieved in all patients. In 97% (34/35) of patients, the seed node was successfully resected. No adverse events occurred during the implantation of the radioactive seed in the axilla, and only one post-operative complication was reported. Our results strengthen the evidence that the specific resection of a previously proven metastatic node combined with SLND confers a clinical advantage. Even when patients have a normal targeted node aspect on post-NAC imaging or a regression in size, they should still undergo TAD, as 57% (12/21) of these patients had residual disease in the axilla. Among patients with complete clinical response on examination and minor abnormal features on imaging, 7/12 (54%) patients had no residual disease in their TAD specimen. The decision to perform TAD in these cases should be carefully made by a multidisciplinary team of oncology experts. Our data suggest that TAD can be a safe option in these patients. Furthermore, we did not experience any difficulty with the concomitant utilization of a radioactive seed in both the axilla and the breast during breast-conserving surgeries. In this study, a significant proportion of patients (50%) avoided ALND. The MD Anderson group reported that ALND was avoided in 55% of cases based on TAD pathology results [15]. In a subsequent study in a larger cohort (n=208), the reported rate was 37% [17], whereas Diego et al. reported rates of 78% [18]. Caudle et al. demonstrated in a prospective study that the FNR of TAD was only 2% [17]. Hence, our purpose was not to reassess the FNR of the procedure. To our knowledge, our group is the first to report on TAD in the Canadian setting and to perform a cost analysis. Based on the current data, we believe TAD should be performed in all centers with access to surgical oncologists and breast radiologists.

Recently, Simons et al. published a multicentric retrospective study on the combination of the MARI procedure and SLND in four hospitals in the Netherlands [19]. We noted important differences in the procedure compared to our study. First, there was a variability in the methodology: a guide wire was inserted in 51.1% of patients rather than an iodine-125 seed [19]. Second, the node was marked with either a clip or a seed only if proven metastatic, implying an additional procedure for all patients. In cases where an iodine seed was used, no clip was inserted. However, having both a clip and a radioactive seed in the targeted node increases the likelihood of successful identification since radioactive seed malposition or displacement can occur.

Simons et al. reported differences in practices for the SLND procedure [19]. A dual-tracer method (technetium-99 and a blue dye) was not always used: one site performed SLND with technetium-99 or blue dye alone [19]. In the present study, we used a dual-tracer method, which is known to decrease the FNR of the procedure [11]. Also, we obtained a radiography of the specimen to ensure proper removal of the targeted node, whereas this was not routinely performed by Simons et al. [19]. Lastly, immunohistochemistry was obtained in all cases in our study based on evidence showing increased detection of micrometastases [9].

Mean hospital costs

Overall, the mean cost of TAD was similar to ALND and SLND performed during the same procedure (4,322 Can$ and 4,479 Can$, respectively). Seed insertion itself did not substantially increase the mean cost of the procedure. We found that TAD was 25% more expensive than ALND for the hospital. However, this is an estimate in a small cohort of patients. The impact of TAD on the total cost of the hospital stay was less significant (20%) since most patients who undergo TAD are not admitted. TAD is usually performed as an outpatient procedure. This represents a main advantage for TAD as well as the lower risk of adverse events compared to ALND. Indeed, the lower risk of lymphedema could potentially decrease the long-term cost of TAD, as most patients do not require physical therapy nor subsequent treatments for postoperative adverse events.

We strongly believe that TAD has more potential qualitative benefits than costs. TAD is a more rapid procedure than ALND and is less complex to perform for the surgical staff. The mean hospital cost of TAD is not prohibitive since it can prevent a second surgery with additional morbidity in a significant proportion of patients. The mean cost of TAD is similar to ALND and SLND performed in one procedure. Three-dimensional intraoperative guidance is provided by the radioactive probe. The pathological analysis of TAD is less time-consuming compared to ALND. Patients are the first group to directly benefit from TAD since it is a less invasive technique with fewer risks than ALND and a higher oncological safety than SLND. However, it involves a supplementary step with potential complications, and patients with residual disease have to undergo subsequent ALND.

Another important consideration is the cost of adjuvant treatments following TAD. Seventeen patients had no residual metastases in their TAD specimen and did not require subsequent ALND. All patients with no residual metastases during TAD were offered adjuvant axillary radiotherapy due to nodal disease on initial diagnosis and lack of ALND. All patients received radiotherapy except for two patients who refused. Despite the fact that these costs are not part of the procedure nor the hospital stay, they have an impact on mid-term and long-term expenditures. Further assessments of these costs in subsequent studies and in larger cohorts should be performed.

Limitations of the study

Our study has some limitations including its retrospective design. Moreover, the size of the cohort affects the power of our statistical analysis. Survival data are needed in order to assess the safety of TAD in this cohort. Also, we did not have access to the cost of the procedure for the patients but only for the hospital. Radiologists in our hospital center have a strong experience with radioactive seed implantation in the breast, which can limit the generalizability of our results.

## Conclusions

Despite higher costs for the hospital, TAD permits to select patients in whom ALND may be avoided post-NAC. Our data demonstrate that ALND can be avoided in 50% of patients in whom TAD is successfully completed. This is a significant proportion of patients in whom the additional morbidity associated with ALND was avoided. We believe that TAD is a safe procedure that should be offered to all admissible patients. The insertion of a clip to mark the biopsy-proven metastatic node is an essential step for the precision of the technique. The long-term impact of ALND omission on patients’ prognosis is under study. With the advent of predictive oncology and artificial intelligence, it will be important to determine how to best identify the subgroup of patients with residual disease in their TAD sampling, who will not benefit from a subsequent ALND.
